# Supplementary material for: UNC119 regulates T-cell receptor signalling in primary T cells and T acute lymphocytic leukaemia
Source: Life Sci Alliance. 2025 Jan 15;8(3):e202403066. doi: 10.26508/lsa.202403066 (PMC11735834; doi:10.26508/lsa.202403066)

Figure 2a – uncropped western blots

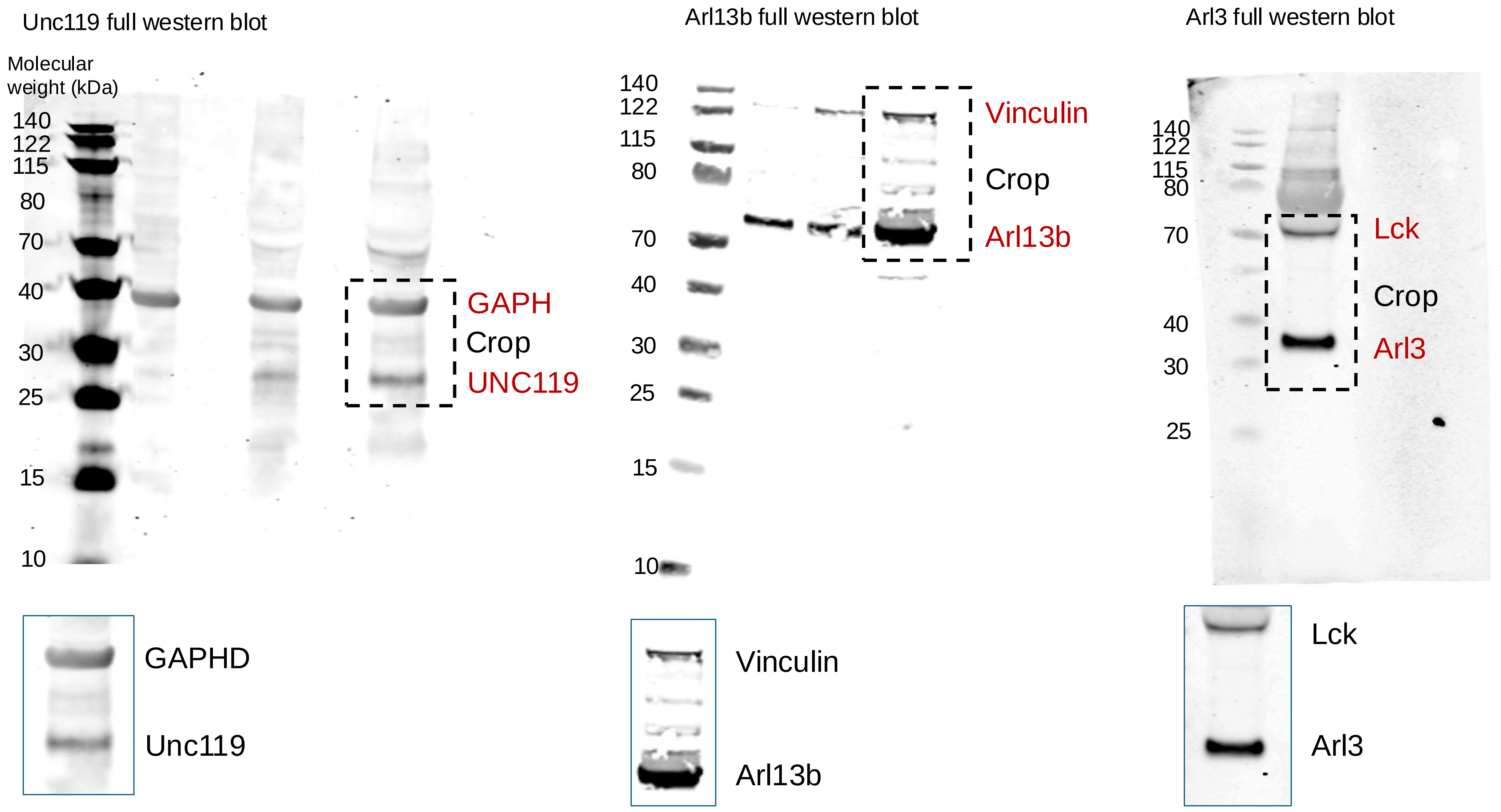

Supplement: Supplementary file 1 [file LSA-2024-03066_SdataF2.pdf]
