# Supplementary material for: UNC119 regulates T-cell receptor signalling in primary T cells and T acute lymphocytic leukaemia
Source: Life Sci Alliance. 2025 Jan 15;8(3):e202403066. doi: 10.26508/lsa.202403066 (PMC11735834; doi:10.26508/lsa.202403066)
Supplement: Supplementary file 2 [file LSA-2024-03066_TableS1.docx]

|  | UNC119A: Squarunkin A complex |
| --- | --- |
| **PDB code** | 9GKG |
| **Data collection** |  |
| Space group | P 21 21 21 |
| Cell Dimensions |  |
| *a, b, c* (Å) | 78.73, 80.63, 191.03 |
| *α, β, γ* (°) | 90.0, 90.0, 90.0 |
| Resolution (Å) | 72.79 – 2.21 |
| R_merge_^a^ | 0.13 (1.53) |
| I/σI | 11.0 (1.1) |
| Completeness (%) | 99.93 (99.98) |
| Redundancy | 6.8 (6.9) |
| CC1/2 | 0.998 (0.628) |
|  |  |
| **Refinement** |  |
| Resolution (Å) | 72.79 – 2.21 |
| No. of reflections | 61,683 |
| R_work_^c^/R_free_^d^ | 20.9/25.4 |
| Bfactors |  |
| Protein | 46.92 |
| Ligand | 36.32 |
| Water | 44.20 |
| R.m.s deviation |  |
| Bond lengths (Å) | 0.013 |
| Bond angles (°) | 1.66 |
| Ramachandran plot statistics |  |
| Favoured region (%) | 96.78 |
| Allowed region (%) | 3.01 |
| Outlier region (%) | 0.21 |

Crystallographic table of statistics

^a^*R*_merge_ = ∑|*I*_obs_ – *I*_avg_|/∑*I*_avg_

^b^The values for the highest-resolution bin are in parentheses

^c^*R*_work_ = ∑|*F*_obs_ – *F*_calc_|/∑*F*_obs_.

^d^Five percent of the reflection data were selected at random as a test set, and only these data were used to calculate *R*_free_
