# Supplementary material for: UNC119 regulates T-cell receptor signalling in primary T cells and T acute lymphocytic leukaemia
Source: Life Sci Alliance. 2025 Jan 15;8(3):e202403066. doi: 10.26508/lsa.202403066 (PMC11735834; doi:10.26508/lsa.202403066)
Supplement: Supplementary file 3 [file LSA-2024-03066_SdataF4.pdf]

Figure 4a – uncropped western blot

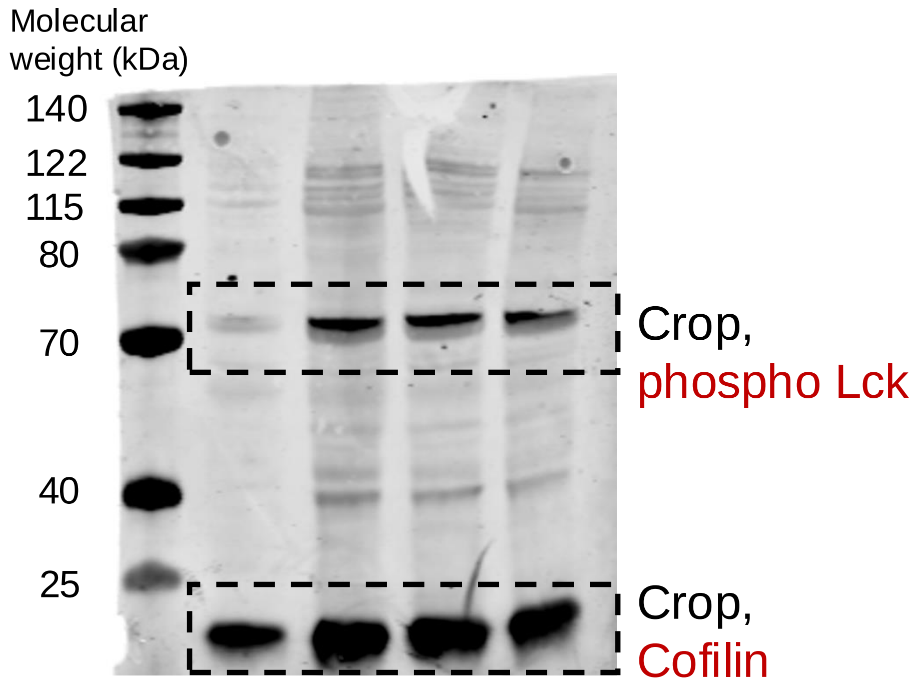

Figure 4b – uncropped western blot

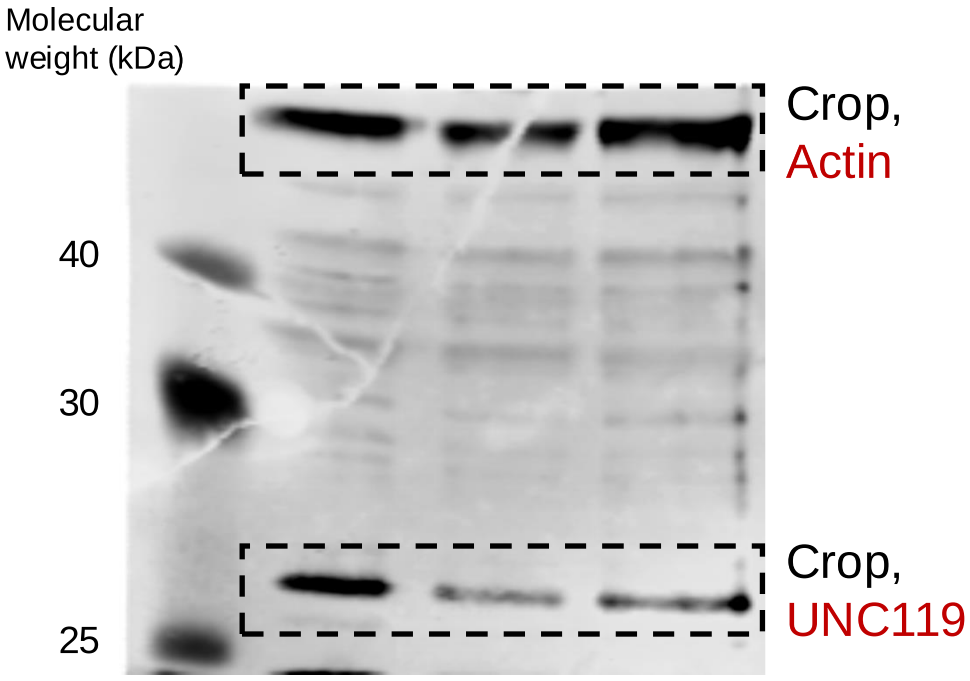

The membrane of this western blot was cut pre incubation with antibodies.
