# Supplementary figures and images for: UNC119 regulates T-cell receptor signalling in primary T cells and T acute lymphocytic leukaemia
Source: Life Sci Alliance. 2025 Jan 15;8(3):e202403066. doi: 10.26508/lsa.202403066 (PMC11735834; doi:10.26508/lsa.202403066)

Figure 5a – uncropped western blot

Molecular  
weight (kDa)

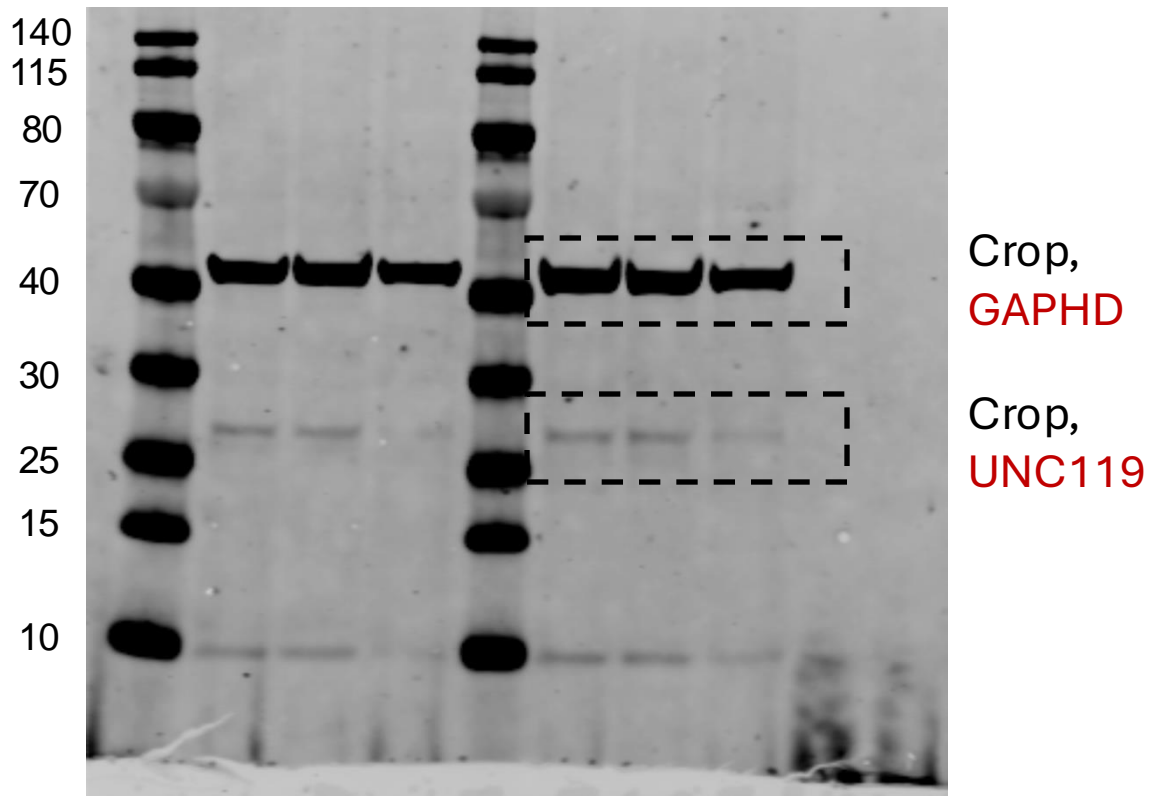

Supplement: Supplementary file 4 [file LSA-2024-03066_SdataF5.pdf]
